# Efficacy and safety of Trans-collateral revascularization of infrapopliteal vessels: A Single-center retrospective study

**DOI:** 10.1186/s42155-025-00595-2

**Published:** 2025-09-26

**Authors:** Yoshinori Tsubakimoto, Jun Shiraishi, Daisuke Usuki, Shin Takiuchi, Satoru Otsuji

**Affiliations:** 1 Department of Cardiology, Higashi Takarazuka Satoh Hospital, 2-1 Nagao-Cho, Takarazuka, Japan; 2 Department of Cardiology, Japanese Red Cross Kyoto Daini Hospital, 355-5 Haruobi-Cho, Kyoto, Japan; 3 Department of Cardiovascular Engineering, Japanese Red Cross Kyoto Daini Hospital, 355-5 Haruobi-Cho, Kyoto, Japan

**Keywords:** Chronic limb-threatening ischemia, Infrapopliteal artery, Chronic total occlusion, Trans-collateral angioplasty, Endovascular therapy, Retrograde approach

## Abstract

**Background:**

Endovascular therapy (EVT) has become a key revascularization strategy for patients with chronic limb-threatening ischemia (CLTI), especially in cases involving infrapopliteal (IP) chronic total occlusions (CTOs), which are often challenging to treat using standard antegrade approaches alone. Trans-collateral angioplasty (TCA) is a retrograde technique that accesses the distal true lumen via collateral vessels when conventional methods are unsuccessful. However, clinical evidence regarding the efficacy and safety of TCA remains insufficient. This study aimed to evaluate the efficacy and safety of TCA as a retrograde approach during EVT for IP CTO lesions.

**Results:**

This retrospective single-center study included 44 IP CTO lesions in patients who underwent TCA between January 2020 and December 2022, after excluding 18 lesions treated solely with the pedal-plantar loop technique. The mean patient age was 78.8 years, and 81.8% had diabetes, 79.5% had chronic kidney disease, and 31.8% were on dialysis. EVT success was achieved in 95.5% (95% CI: 84.9–98.7) of lesions. TCA alone achieved lesion crossing in 70.5% (95% CI: 55.8–81.8), while distal puncture was required in 13.6% (95% CI: 6.4–27.0) of cases. Various crossing techniques, including the rendezvous technique and reverse subintimal tracking, were conducted. Collateral vessel-related complications occurred in 11.3% (5 lesions; 95% CI: 5.0–24.6), including injury in 6.8%, and occlusion and spasm in 2.3%. No vessel dissections occurred. The overall incidence of perioperative complications within 30 days was 20.5% (95% CI: 11.3–34.2), most commonly gastrointestinal bleeding and stroke. At one year, the rate of freedom from target lesion revascularization was 45.4%, and amputation-free survival was 84.0%.

**Conclusions:**

Our findings suggest that TCA can be a feasible and relatively safe retrograde strategy for complex IP CTO lesions when antegrade wiring fails. It is associated with high procedural success and a low incidence of collateral vessel-related complications, supporting its use in selected cases of CLTI.

## Introduction

Chronic limb-threatening ischemia (CLTI) is a severe manifestation of peripheral arterial disease associated with high morbidity, poor prognosis, and a significant risk of limb loss [1]. To improve amputation-free survival (AFS), effective revascularization strategies, such as bypass surgery or endovascular therapy (EVT), are required [2]. However, the selection between bypass surgery and endovascular therapy remains a subject of ongoing debate, even with the publication of recent randomized controlled trials (RCTs) [3, 4]. When EVT is selected, many patients present with complex infrapopliteal (IP) lesions, frequently involving chronic total occlusions (CTOs) classified as Global Limb Anatomic Staging System (GLASS) stage 3 or 4 [5–8]. These CTOs present significant technical challenges due to the difficulty of guidewire passage [9, 10]. In these cases, the failure of the conventional antegrade approach necessitates an alternative strategy, most commonly a retrograde approach involving distal puncture (DP) of a runoff vessel [11–13]. However, DP is not always feasible, particularly in patients where the available runoff vessels are critically stenosed or occluded. Trans-collateral angioplasty (TCA) has developed as a promising technique when standard approaches are not viable [14, 15]. While individual techniques, such as the pedal-plantar loop technique using plantar arch, have been described [16, 17], data on the efficacy and safety of TCA remains insufficient. Moreover, standardized protocols and outcome measures have not been defined. This study aims to evaluate the clinical performance of TCA in EVT for complex IP CTOs in CLTI patients, particularly when conventional retrograde approaches are not viable.

## Methods

### Study Design and Patient Population

**Fig. 1:**
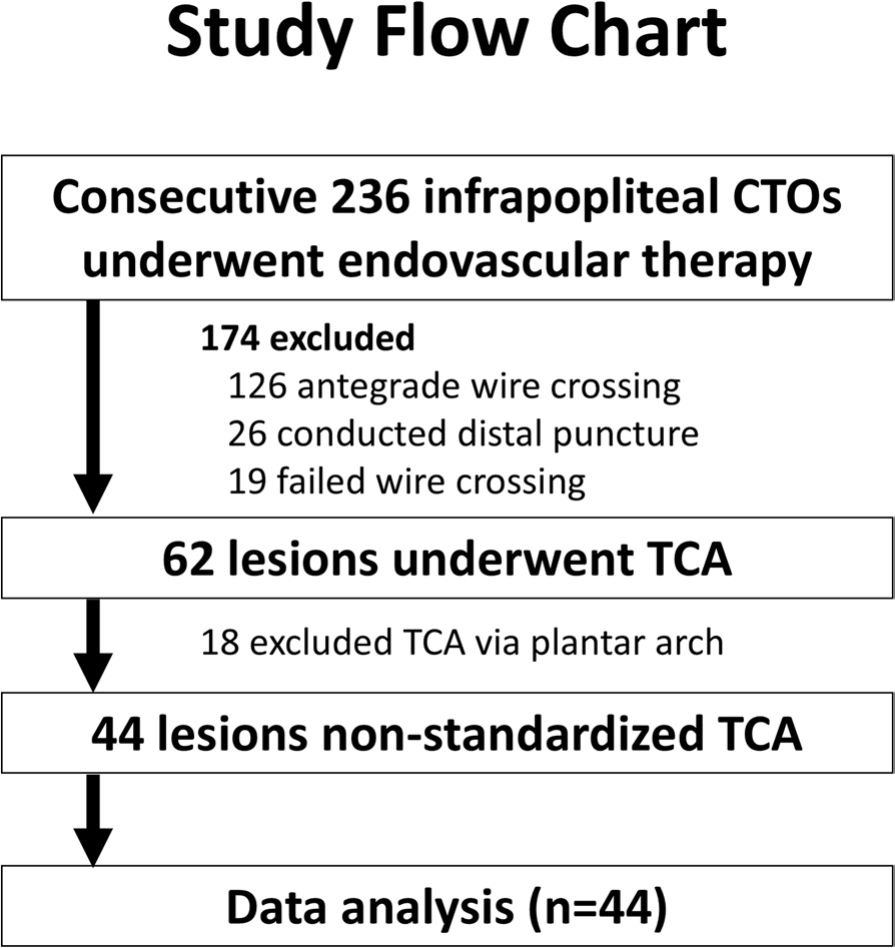
Study Flowchart. CTO, chronic total occlusion; TCA, trans-collateral angioplasty

This was a single-center, retrospective observational study conducted between January 2020 and December 2022. During this period, consecutive 236 IP interventions for CTO were conducted. Of these, 62 IP interventions in 48 CLTI patients underwent TCA due to the failure of antegrade wire crossing. Among these, 18 interventions were performed solely using the pedal-plantar loop technique, which was excluded to maintain focus on non-standardized TCA. Consequently, 44 IP interventions were included in the final analysis (Fig. 1). One month and 12-month follow-ups were scheduled. The study was conducted in accordance with the Declaration of Helsinki, and was approved by the institutional review board.

### Procedure

**Fig. 2:**
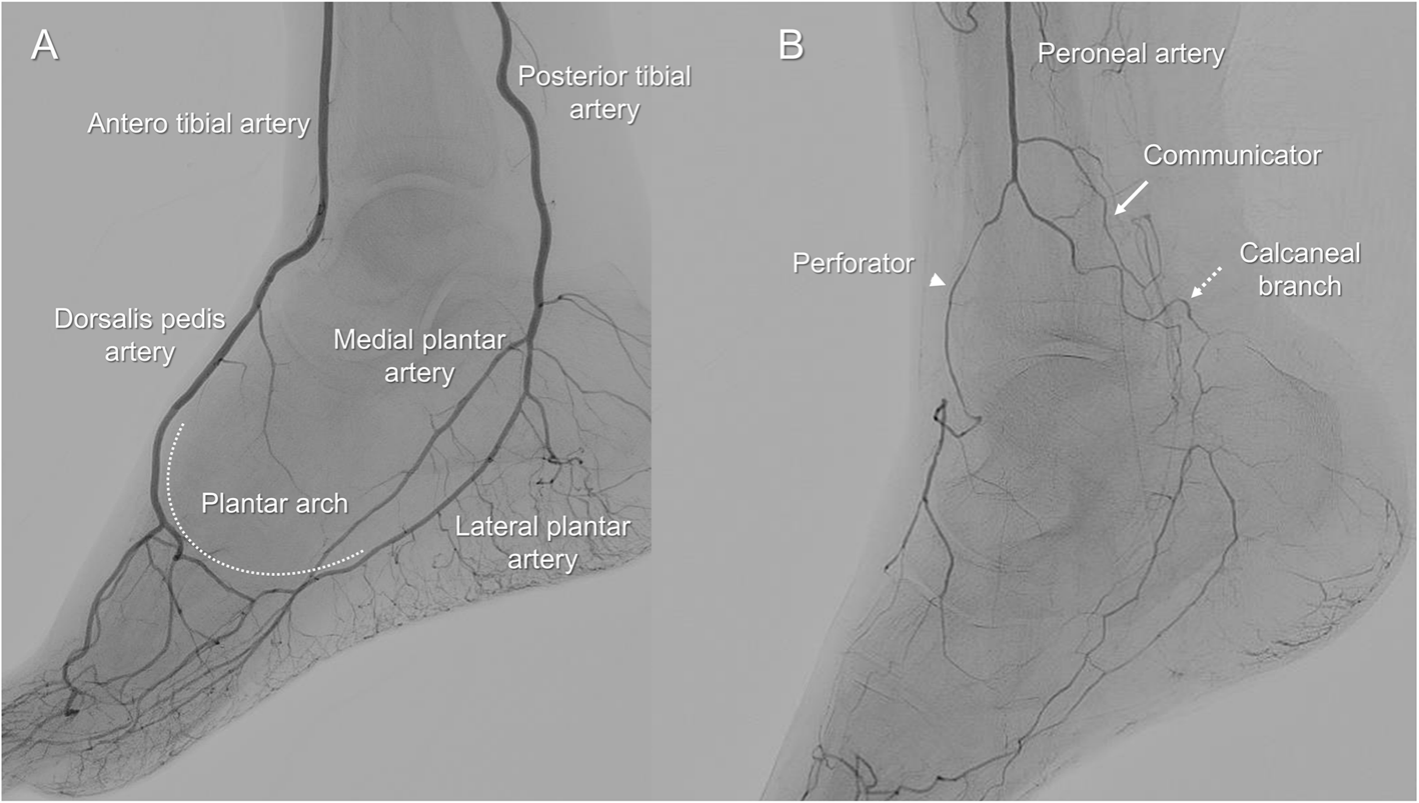
Angiographic images demonstrating the distal foot arterial anatomy relevant to trans-collateral access routes. The left panel shows the plantar arch (dotted line), which connects the dorsalis pedis artery and lateral plantar artery. The right panel highlights three collateral pathways: The perforator (arrowhead), connecting the anterior tibial artery (ATA) and peroneal artery, The communicator (solid arrow), connecting the posterior tibial artery (PTA) and peroneal artery, The calcaneal branch (dotted arrow), forming a collateral connection between the peroneal artery and the common plantar artery

**Fig. 3:**
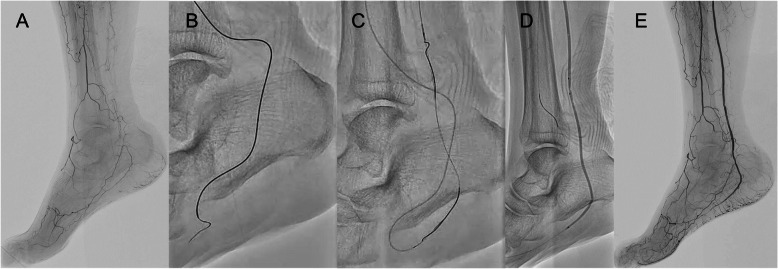
Case 1: a 78-year-old woman with non-healing ulcers on the first and fourth toes of the right foot.** A** Angiography revealed total occlusions of the entire ATA and PTA, as well as the proximal PA in the right IP region. Below-the-ankle runoff was classified as P1 according to the GLASS IM descriptor, and DP was considered challenging in this case. **B** Following successful guidewire passage for PA, tip injection revealed a collateral vessel connecting to the lateral plantar artery was visualized. Therefore, TCA was attempted using this pathway. A Regalia guidewire was carefully advanced into the collateral under the support of a Caravel MC microcatheter. As the distal portion of the vessel was extremely narrow, the wire was switched to an X-treme PV guidewire, which successfully reached the lateral plantar artery. **C** After advancing the microcatheter, the guidewire was exchanged for a Gladius, and retrograde wiring of the PTA CTO was performed. The rendezvous technique was subsequently employed, enabling the antegrade guidewire to cross into the lateral plantar artery. **D** (**E**) Balloon angioplasty was then performed, resulting in satisfactory revascularization on final angiography (Fig. 2**D**, **E**). **A** Initial angiography showing total occlusion of the infrapopliteal arteries with limited below-the-ankle runoff via the medial and lateral plantar arteries

**Fig. 4:**
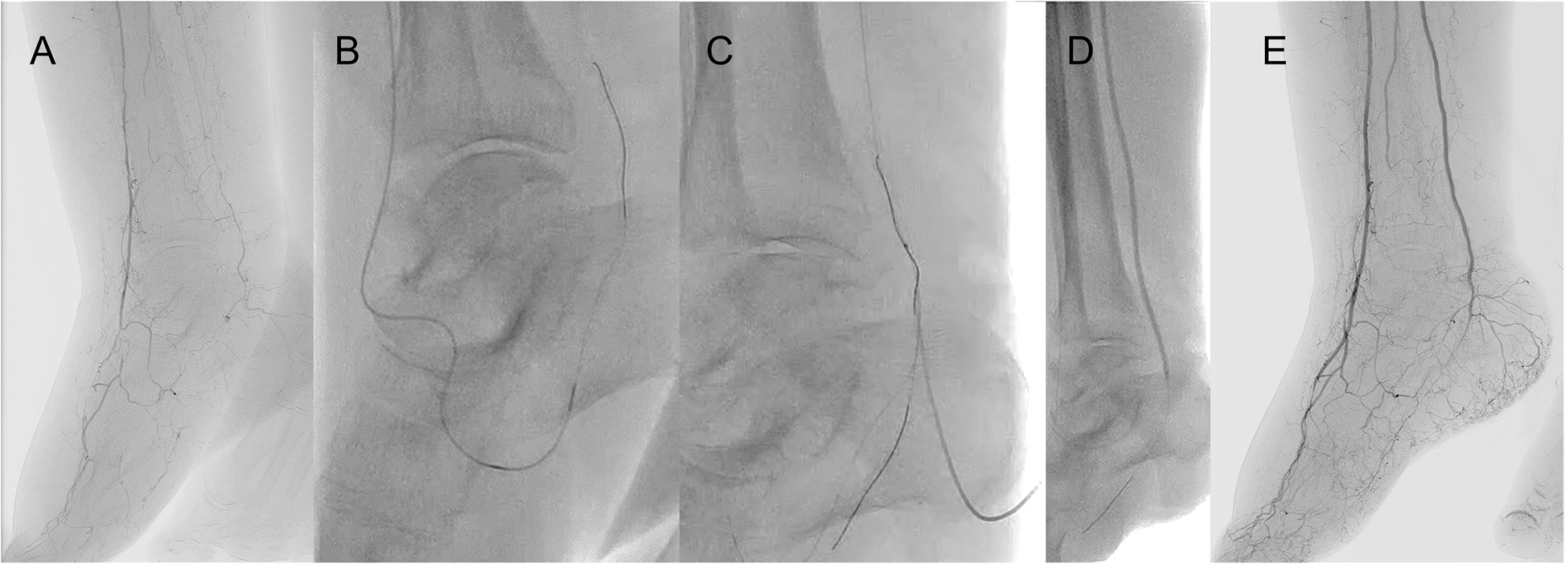
Case 2: a 76-year-old female with type 2 diabetes mellitus, CKD, and CAD was referred to our hospital for a refractory ulcer on the right heel.** A** Initial angiography revealed total occlusions of the distal PA and the whole PTA. Runoff was severely limited, with occlusions of the dorsalis pedis as well as both the lateral and medial plantar arteries, corresponding to a GLASS IM descriptor of P2. **B** As DP was initially deemed unfeasible. TCA was then attempted using a vessel connecting the distal ATA to the partially visualized medial plantar artery. A Regalia guidewire was carefully advanced under the support of a Caravel MC microcatheter, successfully crossing into the distal PTA via the common plantar artery. **C** However, due to the tiny vessel, it was difficult to advance the microcatheter or pass a small-diameter balloon. Therefore, a DP was performed using the retrograde Regalia wire—already advanced into the distal PTA—as a fluoroscopic landmark. A Gladius guidewire was then introduced retrogradely into the PTA, followed by advancement of a Corsair Armet microcatheter. **D** (**E**) Using the kissing wire technique, the antegrade guidewire was successfully navigated into the lateral plantar artery. Subsequent balloon angioplasty achieved direct flow from the PTA to the heel

All angioplasty procedures were performed by interventional cardiologists certified by the Japanese Association of Cardiovascular Intervention and Therapeutics. All patients received dual antiplatelet therapy consisting of aspirin (100 mg/day) and either clopidogrel (75 mg/day) or prasugrel (3.75 mg/day) prior to the procedure. The ipsilateral common femoral artery was used as the access site, with a 4- to 5-French sheath introduced. An initial bolus of 5,000 units of unfractionated heparin was administered via the sheath, followed by additional doses every 30 min to maintain an activated clotting time (ACT) between 250 and 300 s. If femoropopliteal lesions were present, they were treated using optimal balloon angioplasty, with bailout stenting as required, following conventional endovascular techniques. The fundamental principle in target vessel selection for IP lesions is to recanalize the anatomically least challenging artery to achieve in-line flow to the foot [18]. IP occlusive lesions were initially approached using a 0.014-inch guidewire for lesion crossing. If antegrade wire passage was unsuccessful, a retrograde strategy involving TCA was initiated utilizing available collateral pathways identified on the preprocedural angiography. The selection of collateral pathway was based on the discretion of the operator. For reference, the normal anatomy of the lower limb arteries at the ankle level is shown (Fig. 2A). The collateral pathways used for TCA were categorized as follows:

1. Communicator/Calcaneal branch: Communicator connects Peroneal Artery (PA) to posterior tibial artery (PTA) and Calcaneal branch connects PA to common plantar artery. Although often small and tortuous, they may be used when plantar arches are underdeveloped or occluded (Fig. 2B).
2. Perforator: This is an intramuscular or septal pathway that connects PA to anterior tibial artery (ATA) or dorsalis pedis artery. Their identification requires careful angiographic evaluation, and wire manipulation through these vessels is technically demanding (Fig. 2B).
3. Other collateral pathways: This category includes atypical or unnamed collateral pathways not falling into the above classifications, often observed in patients with longstanding chronic total occlusions or severe arterial remodeling. Two representative cases are shown in Figs. 3 and 4.

During TCA procedures, a 0.014-inch Regalia or X-treme PV guidewire (both manufactured by Asahi Intecc, Japan) was carefully advanced into the selected collateral vessel under the backup support of a microcatheter. The guidewire was incrementally advanced with microcatheter follow-up in a stepwise fashion. Upon successful navigation into the distal true lumen of the target artery, retrograde wiring was initiated. If a retrograde wiring via TCA was unsuccessful or inadequate due to poor wire controllability or insufficient backup support, DP was subsequently performed using the retrograde TCA wire as a visual landmark. To achieve lesion crossing, various techniques were employed, including the rendezvous technique, reverse controlled antegrade and retrograde subintimal tracking (r-CART), kissing wire technique, and intravascular ultrasound (IVUS)-guided techniques. After guidewire passage was achieved, balloon angioplasty was performed using an appropriately sized long balloon, maintained for at least 3 min.

**Fig. 5:**
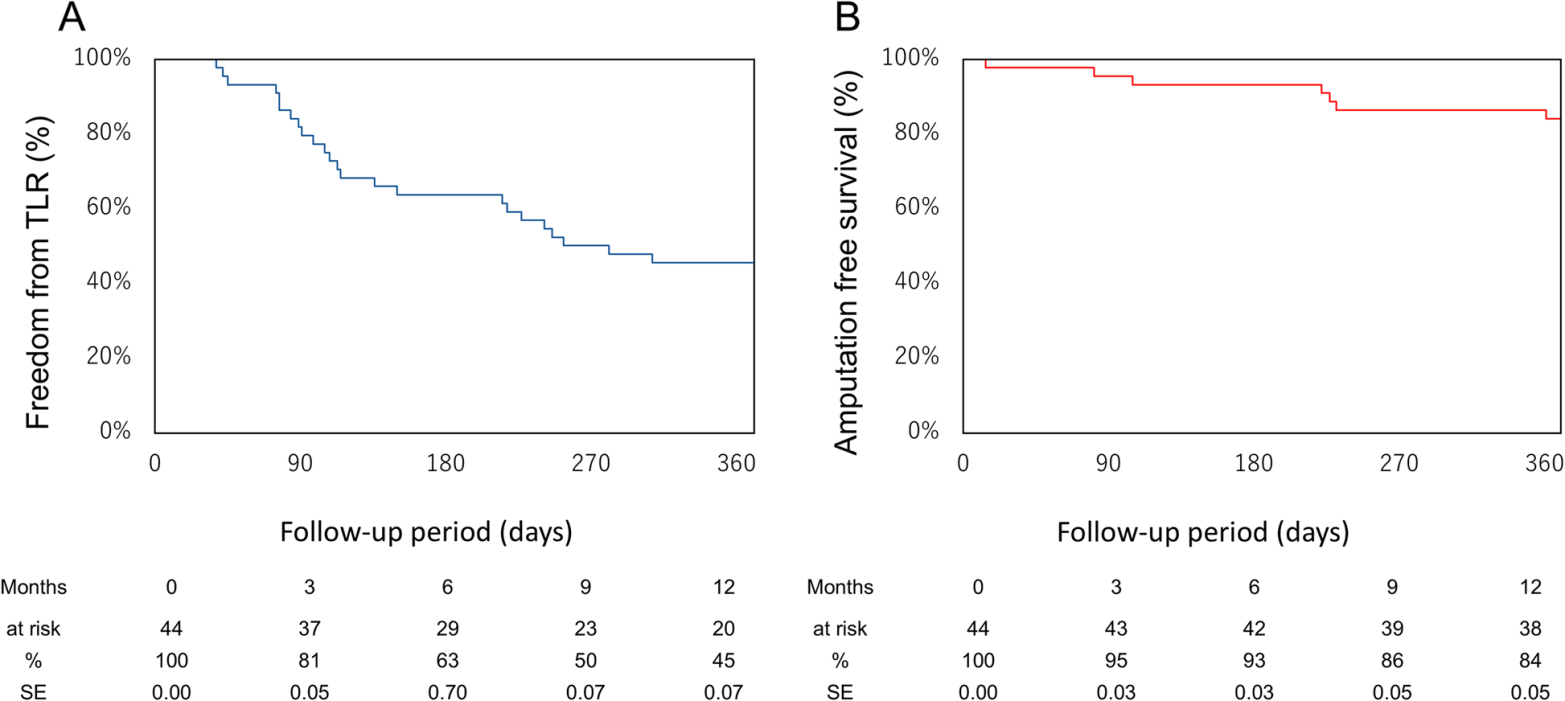
Kaplan–Meier curves for 12-month clinical outcomes after trans-collateral angioplasty (TCA). (A) Freedom from target lesion revascularization (TLR). (B) Amputation-free survival. Below each panel, the number of patients at risk, corresponding survival rates (%), and standard error (SE) at each time point are shown

### Definitions

CLTI was defined based on previous guidelines [2, 18]. Chronic kidney disease (CKD) was defined as estimated glomerular filtration rate (eGFR) of less than 60 mL/min/1.73 m2. Coronary artery disease (CAD) was defined as a history of coronary revascularization, including percutaneous coronary intervention or coronary artery bypass grafting. Cerebrovascular disease (CVD) was defined as a documented history of ischemic stroke and/or intracerebral hemorrhage. The anatomical severity of IP disease was assessed using the GLASS IP grade, while the GLASS inframalleolar (IM) descriptor was used to evaluate the distal runoff. Briefly, the IM grades were defined as follows: P0, an intact pedal arch; P1, a target artery crosses the ankle, but the pedal arch is absent or severely diseased; and P2, no target artery crosses the ankle [2]. Calcification severity was evaluated using the Proposed Peripheral Arterial Calcium Scoring System (PACSS), which grades lesions from 0 (no calcification) to 4 (severe bilateral calcification > 5 cm). PACSS grades 3 and 4 are generally considered indicative of severe calcific burden and may affect procedural planning and outcomes [19]. Amputations were categorized as major (above the ankle) or minor (below the ankle). AFS was defined as survival without the need for major amputation. Target lesion revascularization (TLR) was defined as any repeat percutaneous or surgical revascularization procedure performed to treat restenosis or occlusion within the originally treated segment.

### Outcome measures

The primary outcomes were the EVT success rate, defined as successful EVT of the target CTO with residual stenosis < 30% and restoration of antegrade blood flow regardless of the access route, and the TCA success rate, defined as successful crossing of the target lesion via the collateral pathway followed by revascularization achieved through this pathway alone without the need for distal puncture. Additional primary outcomes included the occurrence of procedure-related vessel complications, such as vessel injury, dissection, occlusion, and spasm, as well as the incidence of perioperative complications (POCs) within 30 days. POCs were defined as adverse events occurring within 30 days of the procedure, including myocardial infarction, stroke, hematoma, gastrointestinal bleeding, initiation of hemodialysis, and the need for blood transfusion [20]. Secondary outcomes included the rates of freedom from TLR and AFS at one year.

### Statistical Analysis:

Continuous variables are expressed as mean ± standard deviation (SD), and categorical variables are presented as counts with percentages and 95% confidence intervals (CI), calculated using the Wilson score interval, unless otherwise specified. Time-to-event outcomes, including freedom from TLR and AFS, were analyzed using the Kaplan–Meier method, and 95% CIs were calculated based on Greenwood’s formula. Follow-up duration was calculated from the date of the index procedure to the occurrence of the event or the date of the last available clinical assessment. Patients without events at the end of the follow-up period were censored. All statistical analyses were performed using JMP Pro, version 17.2.0 (SAS Institute Inc., Cary, NC, USA).

## Results

### Baseline clinical and procedural characteristics

Baseline clinical characteristics in this study were summarized in Table 1. The mean age was 78.8 ± 11.5 years, and 47.7% were male. Comorbidities were common: diabetes mellitus was present in 81.8% of patients, CKD in 79.5%, and 31.8% were undergoing maintenance hemodialysis. Hypertension and dyslipidemia were observed in 77.3% and 38.6% of the cohort, respectively. Additionally, 4.5% were current smokers, 40.9% had a history of CAD, and 6.8% had CVD. Lesion and procedural characteristics are shown in Table 2. The ATA was the most frequently treated vessel (56.8%), followed by the PTA (29.5%) and the PA (9.1%). The mean reference vessel diameter was 2.2 ± 0.3 mm, and the mean lesion length and CTO length were 21.8 ± 3.9 cm and 14.5 ± 10.6 cm, respectively. Based on the GLASS IP classification, the majority of lesions were categorized as grade 3 (43.2%) or grade 4 (36.4%). According to the GLASS IM descriptor, P1 and P2 accounted for 36.4% and 43.2% of cases, respectively. Calcification was assessed using the PACSS, with 38.6% of lesions categorized as PACSS grades 3–4. The distribution of pathways used for TCA was as follows: Perforator (29.5%), Communicator/Calcaneal branch (52.3%), and other collateral pathways (18.2%).

**Table 1:** Baseline patients’ characteristics

Variable	Overall (n = 44)
Age, years	78.8 ± 11.5
Male	47.7 (21)
Diabetes mellitus	81.8 (36)
Hypertension	77.3 (34)
Dyslipidemia	38.6 (17)
CKD	79.5 (35)
Daily hemodialysis	31.8 (14)
Current smoker	4.5 (2)
CAD	40.9 (18)
CVD	6.8 (3)

Continuous values are shown as mean ± standard deviation; categorical data are given as percentage (number)

CKD, chronic kidney disease; CAD, cardiovascular disease; CVD, cerebrovascular disease

**Table 2:** Lesion and Procedural Characteristics

Variable	Overall (n = 44)
Target lesion	
Antero tibial artery	56.8 (25)
Posterior tibial artery	29.5 (13)
Peroneal artery	9.1 (4)
Other	4.6 (2)
Reference diameter (mm)	2.2 ± 0.3
Lesion length (cm)	21.8 ± 7.0
CTO length (cm)	14.5 ± 9.4
GLASS IP grade	
2	20.5 (9)
3	43.2 (19)
4	36.4 (16)
GLASS IM descriptor	
P0	20.5 (9)
P1	36.4 (16)
P2	43.2 (19)
PACCS	
0–2	61.4 (27)
3–4	38.6 (17)
Collateral pathway utilized	
Perforator	29.5 (13)
Communicator/Calcaneal branch	52.3 (23)
Other collateral pathway	18.2 (8)

Continuous values are shown as mean ± standard deviation; categorical data are given as percentage (number)

CTO, chronic total occlusion; GLASS, Global Anatomic Staging System; PACSS, IP, Infra-popliteal; IM, Infra-malleolar; PACCS, Proposed Peripheral Arterial Calcium Scoring System

### Primary outcomes

Primary outcomes are summarized in Table 3. Among the 44 lesions treated, EVT success was achieved in 95.5% (42 lesions; 95% CI: 84.9–98.7). TCA success rate was sufficient in 70.5% (31 lesions; 95% CI: 55.8–81.8), while adjunctive DP with TCA was required in 13.6% (6 lesions; 95% CI: 6.4–27.0) to complete the procedure. In 7 lesions, TCA was attempted but abandoned due to failure of collateral vessel crossing. Techniques of guidewire passage included the rendezvous technique in 27 lesions, the r-CART technique in 1 lesion, the kissing wire technique in 9 lesions, and the IVUS-guided wiring in 3 lesions. Collateral vessel-related complications were relatively rare, with an overall incidence of 11.3% (5 lesions; 95% CI: 5.0–24.6), including vessel injury in 6.8% (3 lesions), occlusion in 2.3% (1 lesion), and spasm in 2.3% (1 lesion). No cases of vessel dissection were reported. The overall incidence of POCs was 20.5% (95% CI: 11.3–34.2). Gastrointestinal bleeding was the most frequently observed complication (13.6%), followed by blood transfusion (11.4%) and stroke (6.8%). No cases of myocardial infarction or puncture site hematoma were reported. Initiation of hemodialysis was required in 2.3%.

**Table 3:** Perioperative Outcomes

Variable	Overall (n = 44)
EVT success	95.5 (42; 95% CI: 84.9–98.7)
TCA success	70.5 (31; 95% CI: 55.8–81.8)
DP combination	13.6 (6; 95% CI: 6.4–27.0)
Wire passage technique	
Rendezvous	61.4 (27)
reverse CART	2.3 (1)
Kissing wire	20.4 (9)
IVUS-guided technique	6.8 (3)
Collateral vessel-related complication	11.3 (5; 95% CI: 5.0–24.6)
Vessel injury	6.8 (3)
Dissection	0.0 (0)
Occlusion	2.3 (1)
Spasm	2.3 (1)
Total POCs	20.5 (9; 95% CI: 11.3–34.2)
Myocardial infarction	0.0 (0)
Stroke	6.8 (3)
Puncture site hematoma	0.0 (0)
Blood transfusion	11.4 (5)
Initiation of hemodialysis	2.3 (1)
Gastrointestinal bleeding	13.6 (6)

Categorical data are given as percentage (number); 95% confidence intervals are provided for key outcomes

EVT, endovascular treatment; CI, confidence intervals; TCA, trans-collateral angioplasty; DP, distal puncture; CART, controlled antegrade and retrograde subintimal tracking; IVUS, intravascular ultrasound; POCs, Perioperative complications

### Secondary outcomes

At one year, the freedom from TLR rate was 45.4% (95% CI: 30.9–59.0), while the AFS rate was 84.0% (95% CI: 70.3–91.8). Kaplan–Meier analysis showed a gradual decline in TLR-free survival over the first year (Fig. 5A), whereas AFS remained relatively stable throughout the follow-up period (Fig. 5B).

## Discussion

This study demonstrates that TCA is a feasible and effective retrograde strategy for managing complex IP lesions, particularly when conventional antegrade wiring techniques are unsuccessful. In this study, TCA success was achieved in 70.5% of cases, contributing to an overall EVT success rate of 95.5%. These results support the clinical feasibility of TCA in real-world settings, with low rates of access-related complications. It is noteworthy that DP, typically the first-line retrograde option, was required in only 13.6% of cases. This low utilization rate demonstrates the utility of TCA in reducing the need for more invasive puncture strategies. Interestingly, TCA can enable DP in cases where it would typically be considered unfeasible due to poor runoff. By navigating a retrograde guidewire through collateral pathways and reaching the distal artery, operators can identify the vessel location visually, thereby providing a reliable landmark for performing DP safely. Therefore, TCA serves not only as an independent treatment strategy but also enhances the applicability of conventional antegrade and/or retrograde techniques in anatomically challenging cases. Despite its benefits, TCA presents technical challenges. Retrograde guidewires navigating through tortuous and/or tiny collateral vessels often exhibit reduced torque response and limited controllability. Moreover, the limited backup force relative to standard retrograde access routes may compromise the ability to cross the lesion. Nevertheless, our findings suggest that the addition of DP can effectively overcome the limitations associated with TCA alone. This synergistic interplay between TCA and DP enhances procedural versatility and may contribute to higher technical success rates. In this study, various collateral pathways were utilized, with communicator/calcaneal branch and perforator routes being the most common. Interestingly, although less frequently utilized, non-standard collateral pathways proved unexpectedly useful in select cases, as illustrated in Case 1 and Case 2 (Figs. 3 and 4, respectively). These findings support the importance of comprehensive anatomical understanding to enhance procedural adaptability. The high prevalence of rendezvous techniques (61.4%) Likely also contributed to the overall EVT success. Importantly, TCA was associated with a relatively low incidence of complications: 6.8% for vessel injuries and no events of vessel occlusion. These findings are comparable to previous reports of IP interventions utilizing retrograde access by distal puncture or using plantar arch access, supporting the safety of the technique when performed by experienced operators with appropriate device selection and techniques [16, 17, 20, 21]. Regarding one-year clinical outcomes, including 45.4% freedom from TLR and 84.0% AFS, are also consistent with previous RCTs [3, 4] or studies in similarly high-risk populations, including those underwent IP interventions requiring retrograde access [21–25]. This finding supports the potential of TCA to achieve durable clinical outcomes, even in patients with advanced comorbidities including diabetes, CKD, and hemodialysis dependence. In many centers in Japan, interventional cardiologists are already familiar with retrograde approaches for coronary CTO lesions, including collateral channel selection and the use of specialized devices. In our institution as well, because of this background, the technical difficulty of TCA in EVT was considered relatively low, and no distinct learning curve effect was observed during the study period. Nevertheless, as operator experience and device technology continue to advance, the role of TCA is expected to expand. Mastery of both TCA and DP, along with their complementary application tailored to anatomical and procedural complexity, may further enhance procedural versatility and improve outcomes in complex IP revascularization.

## Limitations

This study has several limitations. First, it was a retrospective, single-center analysis, which may limit the generalizability of the findings. Second, the sample size was relatively small, and no comparative group was included for direct outcome comparison. Third, the long-term durability of TCA beyond one year remains unknown. Additionally, procedural success may have been influenced by operator experience, given the technical complexity of TCA. Finally, we did not perform multivariable analysis to identify independent predictors of procedural success, complications, or target lesion revascularization due to the limited sample size, which restricts the ability to adjust for potential confounders.

## Conclusions

Our findings suggest that TCA can be a feasible and relatively safe strategy for the endovascular treatment of complex IP lesions. It offers a high procedural success rate with a low incidence of collateral vessel-related complications and may reduce the need for DP. Further prospective, multicenter studies are needed to confirm these findings, define optimal patient selection, technical protocols, and long-term outcomes.

## Data Availability

The datasets used and/or analyzed during the current study are available from the corresponding author on reasonable request.

## Abbreviations

CLTI: Chronic limb-threatening ischemia
AFS: Amputation-free survival
EVT: Endovascular therapy
RCT: Randomized controlled trial
IP: Infrapopliteal
CTO: Chronic total occlusion
GLASS: Global Limb Anatomic Staging System
DP: Distal puncture
TCA: Trans-collateral angioplasty
ACT: Activated clotting time
PA: Peroneal artery
PTA: Posterior tibial artery
ATA: Anterior tibial artery
r-CART: Reverse controlled antegrade and retrograde subintimal tracking
IVUS: Intravascular ultrasound
CKD: Chronic kidney disease
eGFR: Estimated glomerular filtration rate
CAD: Coronary artery disease
CVD: Cerebrovascular disease
IM: Inframalleolar
PACSS: Peripheral arterial calcium scoring system
TLR: Target lesion revascularization
POC: Perioperative complication
SD: Standard deviation
CI: Confidence interval
